# Vancomycin induced cardiac arrest: a case report

**DOI:** 10.1186/s13256-020-02639-8

**Published:** 2021-02-16

**Authors:** Sharad Khakurel, Sangam Rawal

**Affiliations:** 1 Department of Anesthesiology and Intensive Care, National Trauma Centre, National Academy of Medical Sciences, Kathmandu, Nepal; 2 Department of Anesthesiology and Intensive Care, National Trauma Centre, Kathmandu, Nepal

**Keywords:** Intravenous, Vancomycin, Red Man Syndrome, Cardiac arrest

## Abstract

**Background:**

Rapid intravenous administration of vancomycin may manifest with histaminergic responses with clinical features ranging from mild rashes, pruritus and even shock. This case reports of a child, who was accidentally given intravenous vancomycin within minutes and had a cardiac arrest.

**Case presentation:**

A 9-year-old Asian girl who was scheduled for a limb salvage surgery, received vancomycin preoperatively. As a result of rapid infusion of the drug, the patient developed flushing, pruritus and had respiratory distress with hypotension leading to asystole. However, prompt detection and immediate cardiopulmonary resuscitation revived the patient in time following which sound recovery ensued. We recognised inadvertent brisk infusion of vancomycin as the culprit with strong suspicion of Red Man Syndrome.

**Conclusion:**

Red Man Syndrome, though rarely encountered, can always be life threatening. With a surge in the use of vancomycin, adverse effects associated with its use also rises. So a comprehensive knowledge regarding its rationale use, adverse effects and its prompt management in personnel prescribing it, can be life saving.

## Background

Preoperative antibiotic prophylaxis has become a standard practice, in patients who will have an artificial implant or foreign body implanted as part of the procedure [1]. Vancomycin is one such antibiotic that is used for both prevention and treatment of infections caused by gram-positive bacteria predominantly staphylococcal infections, which is commonly encountered in orthopedic practice. However the use of intravenous vancomycin demands extreme caution, as accidental rapid infusion can precipitate Red Man Syndrome (RMS) that can be life-threatening, if not identified and managed aptly. In this case report, we aim to highlight this rare and yet dreadful adverse effect of vancomycin, that can occur in daily clinical practice.

## Case presentation

We recount of a 9-year-old Asian girl diagnosed with Ewing’s sarcoma of left distal tibia. She had received six cycles of chemotherapy that had reduced the size of periosteal lesion compared to few months back. So she was planned for wide resection of the tumor along with reconstruction of her leg. To prevent the risks of implant associated infection, it was decided that the bone cement used during surgery would be impregnated with vancomycin. Following a routine pre-anesthetic assessment, she was cleared for surgery.

A night before the surgery at around 10 pm, an order of prophylactic intravenous vancomycin 500 milligram (mg) in 100 milliliters (mls) of 0.9% normal saline (NS) over 1 hour was carried out in the orthopedic ward. However the drug was accidently infused in about 5 minutes. Soon the patient was restless, complained of epigastric discomfort along with suffocation and feeling of tightness in her chest and neck. There was visible flushing of her face. Within a matter of seconds, she was dyspneic with oxygen saturation falling to 40% along with drop in blood pressure to 40/20 millimeters of mercury (mm Hg). Suddenly her respiration ceased and carotid pulse was impalpable. Immediately chest compression was initiated along with bag and mask ventilation. 0.9% NS was infused rapidly. The patient’s trachea was intubated and ventilation continued. There was return of spontaneous circulation (ROSC) after a minute of chest compressions. Following ROSC, her heart rate was 140 beats per minute in sinus rhythm, oxygen saturation of 99 % with oxygen and blood pressure of 120/90 mm of Hg with Glasgow coma scale (GCS) of E4M5Vt. She was given an injection of pheniramine 10 mg and hydrocortisone 50 mg intravenously. On auscultation of chest, there was decreased air entry bilaterally along with expiratory wheeze that resolved with salbutamol nebulization. She was shifted to Intensive care unit (ICU) where respiration was controlled by mechanical ventilation overnight under sedation. A chest x-ray done was normal. Overnight there were no issues to be addressed and was planned for gradual tapering of sedation. In the morning, she gradually gained full consciousness with normal arterial blood gas analysis and blood reports. The patient’s trachea was extubated following a successful spontaneous breathing trial with T-piece. She was discharged from ICU in the evening with resumption of normal feeding and discharged from the hospital a day after.

The child was admitted again three weeks later and underwent the contemplated procedure uneventfully.

## Discussion

The prescription of antibiotics is inevitable and with it its unwanted adverse effects. Vancomycin use brings to the table the risks of two hypersensitivity reaction namely anaphylaxis and Red Man Syndrome [2, 3]. While it is a daunting task to differentiate anaphylaxis from RMS clinically, the latter is believed to be related to the rate of intravenous infusion and it being the more common reaction between the two [3]. RMS, an anaphylactoid reaction, owes it effects to histamine release following mast cell degranulation whereas anaphylaxis is Ig-E mediated. Role of plasma tryptase to differentiate the two reaction is doubtful. RMS is seen in 3.7 to 47% in infected patients and up to 90% in healthy volunteers [3]. Patients younger than the age of 40, particularly children are vulnerable to its severe form [4]. In children receiving vancomycin, the prevalence of RMS is 14% [5]. The dictum with vancomycin infusion has always been to do so slowly and carefully. However, in our case inadvertent rapid infusion of vancomycin lead to a life threatening situation.

RMS presents with generalized flushing, pruritus and erythematous rash affecting the upper body, neck, and face. In its severe form, dyspnoea, chest discomfort and hypotension can occur. Hypotension without the appearance of a rash has also been reported [6]. RMS is rarely life-threatening, although severe cardiovascular toxicity and even cardiac arrest can occur [6–8]. Our patient had brief flushing of her face with respiratory distress and hypotension which eventually progressed to cardiac-respiratory arrest. We did not look for rashes as cardiopulmonary resuscitation was started immediately and by the time the patient became stable, no flushing and rashes were noted.

RMS is a consequence of histamine release from mast cells and basophils located in the skin, lung, gastrointestinal tract, myocardium, and vascular system [3]. Histamine release occurs as a result of interaction of vancomycin with Mas-Related G-Protein-coupled Receptor X2 (MRGPRX2), a G-protein-coupled receptor mediating Ig-E independent mast cell degranulation [9, 10]. The fact that pretreatment with antihistamines has shown to reduce the effects of RMS further supports the histamine mediated mechanism [11]. The severity of reaction is proportional to the amount of histamine release [3]. Signs and symptoms may occur after 4–10 minutes of intravenous infusion or after its completion and even further delayed presentation have been reported [12].

Although commonly encountered with parenteral use of vancomycin, RMS has also been reported, when used in oral form [13], powder form [13] and vancomycin loaded bone cement [14]. Other antibiotics such as ciprofloxacin, amphotericin B, rifampin, and teicoplanin are also potentially linked with RMS [15]. The concomitant use of opioid analgesics, muscle relaxants, contrast dye with vancomycin seems to augment the effects of RMS [12]. Our patient did not receive any such medications and a link between past use of chemotherapy and vancomycin could not be established.

Vigilance during vancomycin infusion can identify any untoward reaction and warrant intervention. It is recommended to administer intravenous vancomycin over one to two hours duration preferably using volumetric pump with infusion rate not exceeding 10 mg/min [6] Pretreatment with antihistamines may reduce the incidence and severity of RMS [6, 11]. The infusion should be stopped right away when features of RMS is noticed and antihistamines prescribed to treat them. Corticosteroids can come to rescue in refractory cases. Occasionally, there may be the need to maintain circulation with intravenous fluids and ensure adequate oxygenation to stabilize the hemodynamics. Vancomycin desensitization is suggested in patients where the classical treatment modality is unable to stop RMS and there is the absolute need to continue vancomycin [3].

## Conclusion

Vancomycin use in perioperative period is ever flourishing. This report sheds light to a rare adverse effect that can be encountered with its use. However awareness among healthcare practitioners and a well-designed protocol about the vancomycin infusion will prove worthwhile.

## Data Availability

Not applicable.

## Abbreviations

GCS: Glasgow coma scale
ICU: Intensive care unit
Mg: Milligram
Ml: Milliliters
Mm of Hg: Millimeters of mercury
MRGPRX2: Mas-related G-protein-coupled receptor X2
NS: Normal saline
ROSC: Return of spontaneous circulation
RMS: Red Man Syndrome
